# The effect and underlying mechanism of Timosaponin B-II on RGC-5 necroptosis induced by hydrogen peroxide

**DOI:** 10.1186/1472-6882-14-459

**Published:** 2014-12-02

**Authors:** San-Hong Jiang, Lei Shang, Li-Xiang Xue, Wei Ding, Shuang Chen, Ruo-Fei Ma, Ju-Fang Huang, Kun Xiong

**Affiliations:** Department of Anatomy and Neurobiology, School of Basic Medical Science, Central South University, Changsha, Hunan 410013 China; Department of Biochemistry and Molecular Biology, Peking University Health Science Center, Beijing, 100191 China; Xiangya School of Medicine, Central South University, Changsha, Hunan 410013 China

**Keywords:** Retinal ganglion cells-5, Timosaponin B-II, TNF-α, Necroptosis, Oxidative stress

## Abstract

**Background:**

Necroptosis is an important mode of cell death, which is due to oxidant stress accumulation. Our previous study indicated that oxidant stresses could be reduced by Timosaponin B-II (TBII), a kind of Chinese herb *RhizomaAnemarrhenae* monomer extraction. We wonder the possible effect of Timosaponin B-II, whether it can protect cells from necroptosis *via* reducing the oxidant stress, in RGC-5 following hydrogen peroxide (H_2_O_2_) insult.

**Methods:**

RGC-5 cells were grown in DMEM, the model group was exposed in H_2_O_2_ with the concentration of 300 μM, and the experimental group was pre-treated with Timosaponin B-II at different concentrations (1 μM, 10 μM, 100 μM and 1000 μM) for 24 hrs. MTT assay was carried out to measure the cytotoxicity of H_2_O_2_, MDA concentration assay was executed to evaluate the degree of oxidative stress, TNF-α ELISA Assay was used to measure the concentration of TNF-α, finally, the degree of necrosis were analyzed using flow cytometry.

**Results:**

We first constructed the cell injury model of necroptosis in RGC-5 upon H_2_O_2_ exposure. Morphological observation and MTT assay were used to evaluate the degree of RGC-5 death. MDA assay were carried out to describe the degree of oxidant stress. Annexin V/PI staining was used to detect necroptotic cells pre-treated with or without Timosaponin B-II following H_2_O_2_ injury. TNF-α ELISA was carried out to detect the TNF-α accumulation in RGC-5. Upon using Timosaponin B-II with concentration of 100 μM, the percentage of cell viability was increased from 50% to 75%, and the necrosis of cells was reduced from 35% to 20% comparing with H_2_O_2_ injury group. Oxidant stress and TNF-α was reduced upon injury which decreased the ratio of RGC-5 necroptosis.

**Conclusion:**

Our study found out that Timosaponin B-II might reduce necroptosis *via* inhibition of ROS and TNF-α accumulation in RGC-5 following H_2_O_2_ injury.

## Background

Necroptosis is a novel kind of cell death which has similar morphological features of necrosis [1, 2]. The previous studies pointed out that necroptosis was usually initiated by TNF-α or FasL, *etc*. and transmitted by certain specific cytokines, which results in the accumulation of oxidative products and eventually leading to necrosis. However, it can be blocked by necrostatin-1 (Nec-1, a specific inhibitor of necroptosis) [1]. Necroptosis existed in different cell types, such as epidermal keratinocytes [3], Jurkat T cells [4], L929 cells [5, 6], HepG2 cells [7], hippocampal neurons [8], cortical neurons [9] and photoreceptor cells [10], *etc.* upon injury. Furthermore, Rosenbaum, *et al*. have found a large number of necrotic cells exist in rat retinal ganglion cell layer by PI staining at the early stage of acute high intra-ocular pressure (aHIOP) [11]. The numbers of PI-positive neuron decreased, and the "b" wave was partially restored in flash ERG with Nec-1 pretreatment. Dvoriantchikova’s recent works showed that retinal ganglion cells (RGCs) necroptosis promoted in aHIOP-induced retinal damage [12], which has the similar finding with our previous work [13]. Additionally, our recent studies have also indicated that necroptosis occurs in retinal ganglion cells-5 (RGC-5) at an early stage following elevated hydrostatic pressure (EHP) *in vitro*[14] or hydrogen peroxide (H_2_O_2_) treatment (our unpublished data) detected by flow cytometry (a typical way to monitor necroptosis) [15].

**Figure 1:**
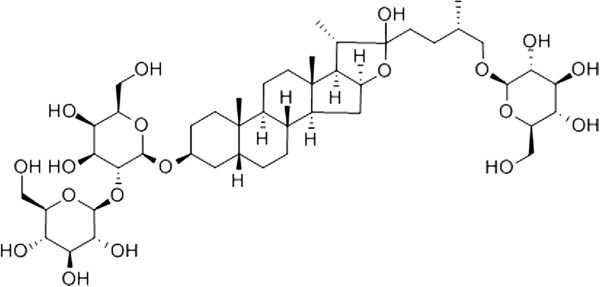
**The chemical structure of Timosaponin B-II.**

Although some scientists recently have synthesized necroptosis inhibitors, such as Nec-3 [16], Nec-5 [17], Nec-7 [18], Nec-21 [19], *etc.,* the mechanism still needs to be further clarified. Nec-1 is a synthetic small molecular compound which is the most commonly used in necroptosis inhibition [1]. So far, there are no reports about whether it has side effects when it is applied to animal or cellular models. Traditional Chinese herb *rhizomaanemarrhenae* is the dried rhizome of *anemarrhenaasphodeloidesbge*. It contains lots of steroidal saponins including sarsasapogenin, markosapogenin, negitogenin, diosgenin and its glycosylated derivatives, like Timosaponin A-I, A-II, A-III, A-IV, B-I, B-II,C, D and Timosaponin E [20]. Li [21] indicated that Timosaponin B-II (Figure 1, the chemical structure of Timosaponin B-II, CAS number: 136656-07-0) might improve the impairment of learning and memory caused by cerebral ischemia in a dose-dependent manner. Lu [11] and Kim’s [22] research suggested that, Timosaponin B-II could not only inhibit the production of IL-1β and IL-6, but also TNF-α (one of important molecules which initiates necroptosis), these results suggested the effect might be related to Timosaponin B-II which has anti-inflammatory activity. Besides, Zhang [23] and Kaname [24] found Timosaponin E-I, E-II, B-II, B-III and A-III played an important role in superoxide dismutase-generation. Deng [25] demonstrated that culturing primary rat neurons (Aβ_25–35_ insult model) with Timosaponin B-II at a certain range of concentrations showed remarkable anti-oxidative damage effects, which indicated Timosaponin B-II could remove oxygen radicals, and keep the intra-cellular redox reactions in a dynamic equilibrium [26]. Our previous study also showed that Timosaponin B-II participated in the protection of rat RGCs which was treated by FeCl_3_ solution *via* anti-oxidation [27]. Of note, whether Timosaponin B-II could inhibit oxidative stress-induced RGCs necroptosis or not, it is still under investigation. Therefore, our present experiments focused on Timosaponin B-II’s potential roles in RGC-5 necroptosis suppression upon hydrogen peroxide (H_2_O_2_) treatment. Meanwhile, the effect of Timosaponin B-II on malondialdehyde (MDA, biomarker of oxidative stress) and TNF-α in RGC-5 has been investigated. Our study may give a better understanding of the protective effect of Timosaponin B-II upon neuronal injury, and provide the experimental basis of novel mechanism on cell death.

## Methods

### Cell culture

The mouse retinal ganglion cell line (RGC-5) was contributed by Department of Ophthalmology, Second Hospital of Ji Lin University, China [28]. RGC-5 cells were grown in Dulbecco’s Modified Eagle Medium (DMEM, HyClone Laboratories, Inc. UT, USA) and supplemented with 10% fetal bovine serum (FBS, HyClone Laboratories, Inc. UT, USA), 100 U/ml of penicillin and 100 μg/ml of streptomycin (HyClone Laboratories, Inc. UT, USA). The RGC-5 cells used in the experiment were with 2-3 passages post-thawed to minimize the variability in the assays based on our observations. The density of RGC-5 cells was around 80% in 6 ml culture media in 50 ml flask before insults (H_2_O_2_ treatment).

### Reagent

The powder of Timosaponin B-II was provided by Professor Wan-Sheng Chen from Department of Pharmacology, School of Pharmacology, Second Military Medical University, Shanghai, China [29, 30] and its purity is above 98%. Timosaponin B-II was dissolved in sterile normal saline (NS) at 2 mM in room temperature (24°C), H_2_O_2_ (Sigma-Aldrich, MO, USA) with 0.01 M PBS at 3 mM in store to yield a low concentration working solutions.

### Cell model construction and drug treatment

Cells were equally divided into three groups randomly. The normal control group (CTL), the model group (H_2_O_2_, 300 μM), and the experimental group (H_2_O_2_ + Timosaponin B-II). The experimental group was pre-treated with Timosaponin B-II at different concentrations (1 μM, 10 μM, 100 μM and 1000 μM) for 24 hrs. No drugs were exposed to the normal control or the model group. After that, we used H_2_O_2_ of 300 μM for 12 hrs to get cell insult and randomly selected three in each group for morphological studies while the remaining cells were used for biochemistry studies. For morphological study, the pictures of the attached cells were captured using inverted microscope in 10 × objective (Olympus, CKX41, Tokyo, Japan). We have captured ten pictures at least in each group, and selected the typical graphics to illustrate.

### MTT assay

Cytotoxicity of H_2_O_2_ model group and Timosaponin B-II pretreated group (1 μM, 10 μM, 100 μM and 1000 μM) were assessed in RGC-5 cells by measuring the amount of insoluble formazan formed in live cells based on the reduction of 3-(4, 5 dimethylthiazol-2-yl)-2, 5-diphenyltetrazolium bromide (MTT) salt (Nanjing Jian-Cheng Bio-engineering Institute, Jiangsu, China) according to the manufacturer's protocol. The cells were seeded in 96-well plates with a density of 10^4^ cells/well incubated for 24 hrs at 37°C and 5% CO_2_. The cells were pretreated with different concentrations of Timosaponin B-II before H_2_O_2_ insult or H_2_O_2_ used alone and PBS as a negative control. Within 24 hrs pre-treatment of Timosaponin B-II, 50 μl of MTT labeling reagent (2 μg/ml) was added to each well. The plates were incubated at 37°C in a humidified atmosphere with 5% CO_2_ for 4 hrs. Thereafter, 100 μl of the solubilization solution was added to each well and followed by incubation overnight at 37°C to dissolve formazan crystals. Absorbance was ultimately read using an ELISA plate reader (Bio-tek, ELx800, IL, USA) at a wavelength of 570 nm. Where, H_2_O_2_ model group and Timosaponin B-II pretreated group are mean absorbance of treated cells and negative control, respectively.

### MDA concentration assay

MDA levels in RGC-5 extractions were assayed using a commercial kit according to manufacturer’s instructions (Nanjing Jian-Cheng Biotechnical Co., Jiangsu, China) as in our previous study [27], the standard reference substance named tetraethoxypropane were used in 10 nmol/ml. Equal quantities (100 μg) of protein were loaded in each well and each analysis performed in duplicate.

### TNF-α ELISA assay

A RGC-5 TNF-α concentration assay was performed using a commercial TNF-α ELISA kit (Invitrogen, CA, USA). The detailed processes were conducted according to the manual included in the kit. Positive control: the antibody tested in the kit was replaced by mouse TNF-α (provided in assay, concentration: 720 ng/L). Equal quantities of protein (80 μg) were analyzed in every tested well and the measurements were carried out by Bio-tek microplate-reader (ELx800, IL, USA). The percentage of TNF-α concentration in normal control group was set as 100%. All experiments were repeated at least twice.

### Flow cytometry

The cells attached to the flasks were trypsinized followed by a gentle wash. The experimental group was pre-treated with Timosaponin B-II at 100 μM. The model group was treated with H_2_O_2_ at 300 μM. Resuspended the cells in 200 μl of 1× binding buffer, and then added 5 μl of 20 μg/ml Annexin V and 10 μl of 50 mg/ml PI, incubated at RT for 15 mins in the dark. After the cells were washed and analyzed by FACS Calibur (Becton, Dickinson Company, NJ, USA). The percentages of cells in each quadrant were analyzed using ModFit software (Verity Software House Topsham, NJ, USA). Statistical results of flow cytometry were conducted by calculating the PI+ cells numbers. All the results were repeated three times.

### Data analysis

Figure panels were assembled by using Photoshop CC (Adobe, CA, USA). The data were analyzed by using SPSS 19.0 (SPSS, IL, USA). One-way analysis of variance (one-way ANOVA) was performed to test differences in average value between groups. All results were presented as mean ± SD. A value of *p* < 0.05 was considered statistically significant.

## Results

### Timosaponin B-II could protect RGC-5 from H_2_O_2_ injury

**Figure 2:**
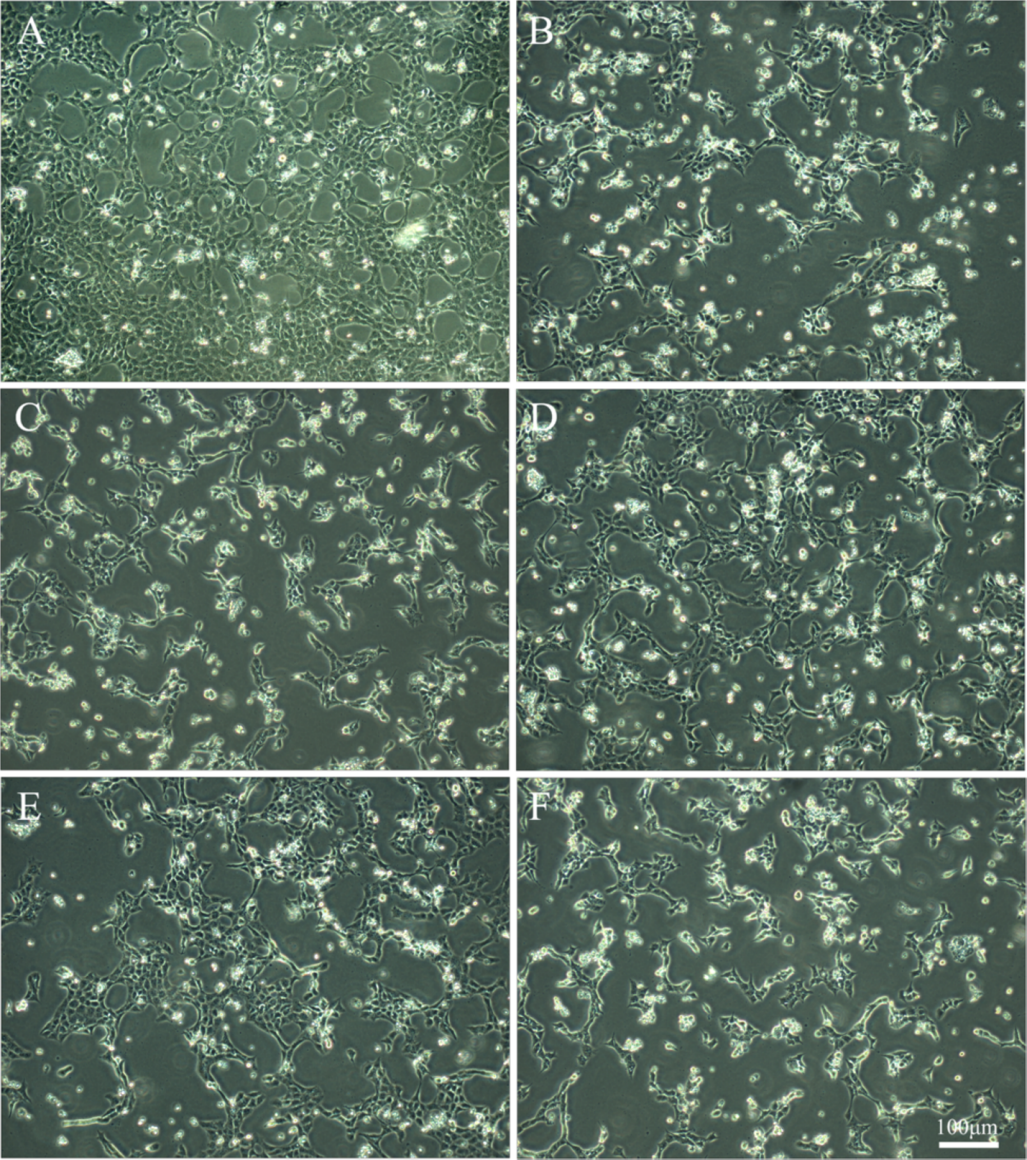
**The morphological graphic of RGC-5 cell line insult with H**_**2**_ **O**_**2**_ **after 12 hrs pretreating in different concentration of TBII by inverted microscope; A: Normal Control; B: Model group (H**_**2**_ **O**_**2**_ **treatment); C: TBII pretreatment group (H**_**2**_ **O**_**2**_ **+ 1 μM TBII); D: TBII pretreatment group (H**_**2**_ **O**_**2**_ **+ 10 μM TBII); E: TBII pretreatment group (H**_**2**_ **O**_**2**_ **+ 100 μM TBII); F: TBII pretreatment group (H**_**2**_ **O**_**2**_ **+ 1000 μM TBII), Scale bar = 100 μm in A-E.**

The normal RGC-5 cell graphic were shown in Figure 2A. The cells grew in apposite density and adherent well with axon-elongated and interweave each other to be a mesh, each of them presented as identical size and consistent morphology. The number of the cells decreased in H_2_O_2_ injury group (Figure 2B), and plenty of them were detached and floated in culture media. As shown in Figure C-E, the number of adherent cells increased following the incubated concentration of Timosaponin B-II increase, it is worth noting that the number of adherent cells in 1000 μM Timosaponin B-II treatment group is less than those in 100 μM Timosaponin B-II treatment group (Figure E and F). These results indicated that Timosaponin B-II could protect RGC-5 from H_2_O_2_ insult yield to the concentration manner, but the effect may reverse with higher concentrations of Timosaponin B-II in 1000 μM.

### Timosaponin B-II could increase RGC-5 viability upon H_2_O_2_ injury

**Figure 3:**
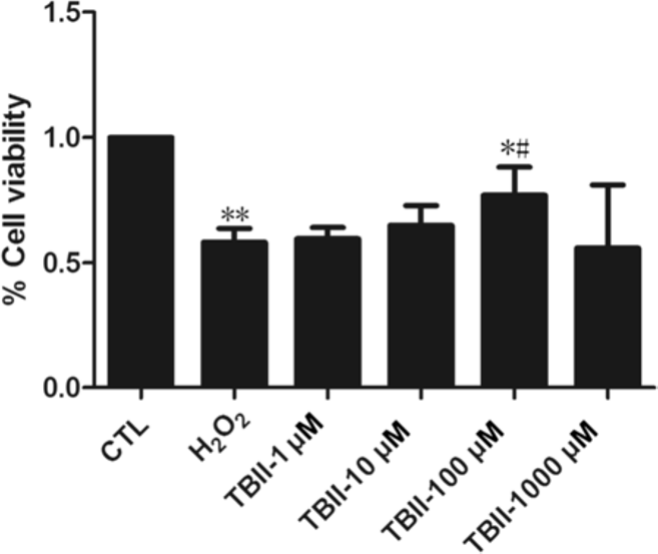
**The percentage of MTT in RGC-5 cell line insult with H**_**2**_ **O**_**2**_ **after 12 hrs pretreating in different concentration of TBII (Normal Control were set as 100%).** **vs CTL: *p* < 0.01; ^#^ *vs* Model group: *p* < 0.05; *vs CTL: *p* < 0.05.

In order to evaluate the viability of RGC-5 in model group and Timosaponin B-II treatment group, we carried out MTT assay to detect cell viability. The statistical analysis of MTT result was shown in Figure 3. Comparing to normal control group (CTL), the cell viability was significantly decreased in model group and Timosaponin B-II treatment group. The cell viability kept gradually increasing upon elevating the concentration of Timosaponin B-II except 1000 μM Timosaponin B-II group. These results indicated that Timosaponin B-II could rescue RGC-5 viability, which was impaired by the H_2_O_2_.

### Timosaponin B-II reduced MDA production in RGC-5

**Figure 4:**
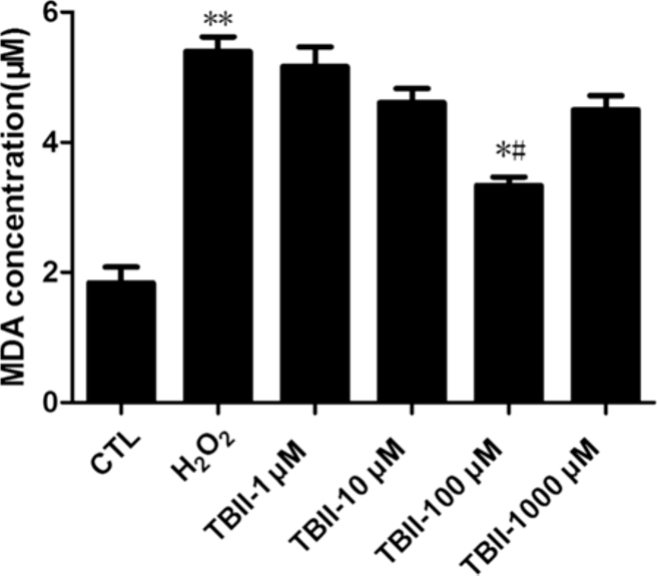
**The concentration of MDA in RGC-5 cell line insult with H**_**2**_ **O**_**2**_ **after 12 hrs pretreating in different concentration of TBII.** **vs CTL: *p* < 0.01; ^#^ *vs* Model group: *p* < 0.05; *vs CTL: *p* < 0.05.

The statistical analysis of MDA level is shown in Figure 4. The level of MDA in the experimental group (except 1000 μM Timosaponin B-II treatment group) kept decreasing gradually compared to the model group upon H_2_O_2_ insult after increasing the concentration of Timosaponin B-II, but it remained at higher level than those in normal control group (CTL). In 100 μM Timosaponin B-II treatment group, the level of MDA decreased significantly compared with H_2_O_2_ insult group (*p* < 0.05). These results indicated that Timosaponin B-II reduced the oxidative stress which is usually induced by H_2_O_2_ in RGC-5. Nevertheless, the capacity of anti-oxidant was limited, because the oxidative stress remained higher than normal control group in Timosaponin B-II treatment group.

### Timosaponin B-II inhibited TNF-α production in RGC-5

**Figure 5:**
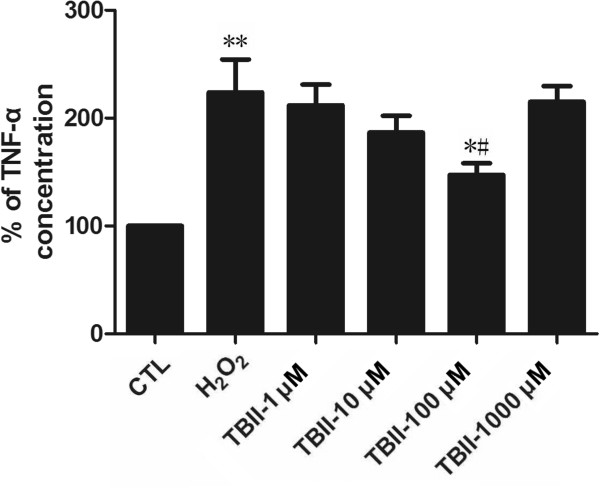
**The percentage of TNF-α concentration in RGC-5 cell line insult with H**_**2**_ **O**_**2**_ **after 12 hrs pretreating in different concentration of TBII.** **vs CTL: *p* < 0.01; ^#^ *vs* Model group: *p* < 0.05; *vs CTL: *p* < 0.05.

The statistical analysis of ELISA assay for TNF-α level was described in Figure 5. The level of TNF-α in the experimental group (except 1000 μM Timosaponin B-II treatment) was gradually decreasing compared to the model group after increasing the concentration of Timosaponin B-II, but it remained at higher levels compared to those in normal control group. These results indicated that Timosaponin B-II remarkably inhibited TNF-α production. Nevertheless, the effect of Timosaponin B-II was impaired to a certain extent since the level of target peptides was still higher than those in normal condition.

### Timosaponin B-II may decrease the rate of RGC-5 necrosis

**Figure 6:**
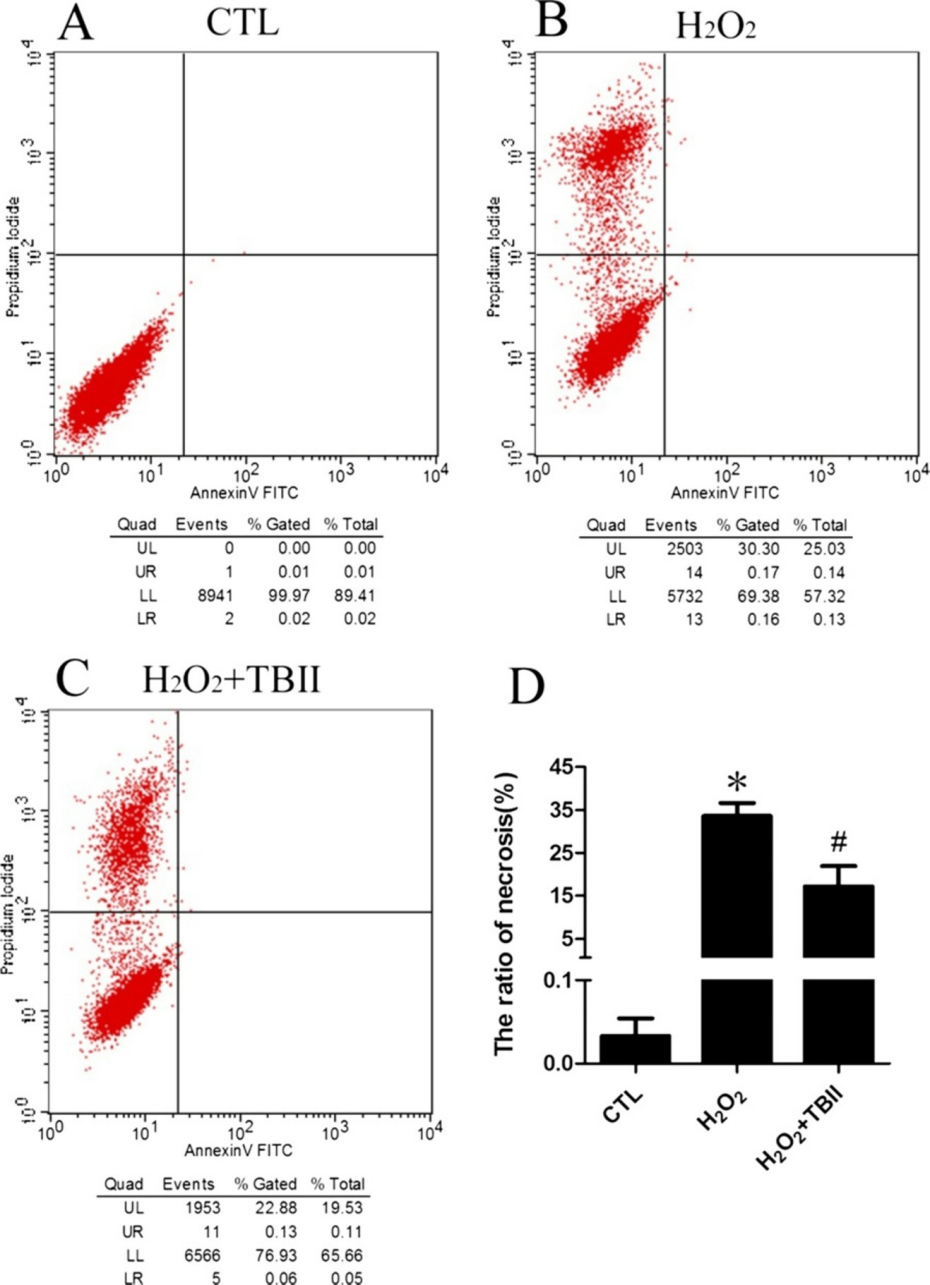
**The flow Cytometry analysis of RGC-5 cell line insult with H**_**2**_ **O**_**2**_ **after 12 hrs pretreating in different concentration of TBII. A**: Normal Control; **B**: Model group (H_2_O_2_ treatment); **C**: TBII pretreatment group (H_2_O_2_ + 100 μM TBII); **D**: Statistical analysis; *vs CTL: *p* < 0.01; ^#^ *vs* Model group: *p* < 0.01.

Upon 300 μM H_2_O_2_ insult, necrosis in RGC-5 occurred in our study. Therefore, the cells were treated under this condition with the addition of Timosaponin B-II at different concentrations. After that, we analyzed cellular necrosis by using flow cytometry with PI/Annexin V double staining, and detected whether it could decrease the rate of necrosis in RGC-5 with pretreatment of Timosaponin B-II upon H_2_O_2_ insult. These results showed that the ratio of necrosis cells is 30.3% (Figure 6B), the percentage decreased to 22.8% upon adding Timosaponin B-II (100 μM, Figure 6C). Meanwhile, statistical analysis indicated that there were significant changes in the rate of PI-positive RGC-5 in Timosaponin B-II pretreatment group compared with normal control group and H_2_O_2_ model group (Figure 6D). These results indicated that RGC-5 necroptosis in the early stage may decrease with Timosaponin B-II usage.

## Discussion

At present, mechanism of cell death is one of the hotspot in the field of life science research. As far as we are concerned, compared to apoptosis and autophagy, the researchers did not pay enough attention to necrosis due to the traditional viewpoint that it cannot be modulated and intervened. Necroptosis is the latest cell death mode which was conceptualized in last century in mid-1980s [31]. Since then, it started to catch more and more attention [32–34]. We speculate that damaged cells could be rescued if necroptosis can be intervened at an early stage, and it will also help us find a better strategy for rational interventional therapy in the future.

Chinese herb has a wealth of resources from natural materials with lower side effects, more economical and other advantages, *etc.* However, it also has the disadvantages such as its complicated composition which cannot be identified easily. Timosaponin B-II is a monomer which is extracted from Chinese herb *rhizomaanemarrhenae*, it has multiple pharmacological effects, including anti-inflammatory [21, 35], anti-diabetic [36], anti-oxidative stress [23, 26, 27], anti-senile dementia [37], learning ability or memory improvement [21] and neuro-protection [27] or anti-apoptosis effect in human umbilical vein endothelial cells [38], *etc*. Our MTT assay results showed that viability of RGC-5 in model group (H_2_O_2_ treatment) was significantly decreased, while RGC-5 was pre-incubated by Timosaponin B-II (1 μM, 10 μM and 100 μM groups) for 24 hrs, RGC-5 proliferation increased gradually in a dose-dependent manner. The RGC-5 proliferation of 100 μM treatment group reached a peak, but it significantly decreased in 1000 μM treatment group. The morphological results suggested that the number of RGC-5 decreased remarkably in model group, the survival of cells tends to be similar as MTT when pretreated with Timosaponin B-II. Taken all together, it indicated that Timosaponin B-II played a protective role on RGC-5 in H_2_O_2_-induced damage under a certain concentration. In addition, our results in flow cytometry showed that the number of RGC-5 cell necrosis significantly reduced in 100 μM Timosaponin B-II pretreated group, which suggested that Timosaponin B-II may be partly involved in inhibiting RGC-5 necroptosis under H_2_O_2_ exposed conditions. Moreover, our results on oxidation products measurement also showed that Timosaponin B-II could significantly decrease the accumulation of MDA in RGC-5 in certain range of concentrations. Furthermore, we had observed that TNF-α was also inhibited in Timosaponin B-II pretreatment group. Considering one of the most important pathways in necroptosis (TNF-α induced necroptosis pathway initiate-----necroptosis related molecular modulation-----ROS accumulation------cell necrosis [39, 40]), we speculated that Timosaponin B-II might inhibit the RGC-5 necroptosis by reducing oxidation products and TNF-α level.

Finally, it is worthy of noting that our experiments revealed the following two questions. Firstly, although Timosaponin B-II can reduce necroptosis by resisting oxidative stress and reduce pro-inflammatory molecule, but it is impossible to recover to normal level, which indicates its limited function as monomer herbal extraction, it also indirectly supports the compatibility usage of Chinese herbal drugs. Secondly, our results showed that higher concentrations of Timosaponin B-II (*e.g*. 1000 μM) could be toxic. Frank’s research showed Timosaponin B-II and Timosaponin A-III have the similar structure (such as steroid), but Timosaponin B-II presents one more glycosyl [41]. Timosaponin B-II has the trend to convert into Timosaponin A-III. However higher concentrations of Timosaponin A-III has been regarded as highly cytotoxic [41–43] and could cause cell death instead of protective effect. Therefore, we would consider discarding this concentration (1000 μM) in future studies. Until now, the molecule of regulation mechanism for cellular necroptosis upon injury was concerned, including receptor interacting proteins (RIPs) [8, 10, 12, 13], calpains [14, 44], CDGSH iron-sulfur domain-containing protein 1 (CISD1) [45] or ubiquitin C-terminal hydrolase (UCH-L1) [46], *etc*. Therefore, whether the possible pathway of Timosaponin B-II neuro-protection may mediate by these molecules mentioned above needs further investigation.

## Conclusion

Timosaponin B-II with limited concentrations can partially protect RGC-5 from H_2_O_2_ induced-necroptosis, which may related to TNF-α and ROS accumulation inhibition.
